# Stillbirth maternity care measurement and associated factors in population-based surveys: EN-INDEPTH study

**DOI:** 10.1186/s12963-020-00240-1

**Published:** 2021-02-08

**Authors:** Lydia Di Stefano, Matteo Bottecchia, Judith Yargawa, Joseph Akuze, M. Moinuddin Haider, Edward Galiwango, Francis Dzabeng, Ane B. Fisker, Bisrat Misganaw Geremew, Simon Cousens, Joy E. Lawn, Hannah Blencowe, Peter Waiswa, and the Every Newborn-INDEPTH Study Collaborative Group, Peter Byass, Stephen Tollman, Hagos Godefay, Joy E. Lawn, Peter Waiswa, Hannah Blencowe, Judith Yargawa, Joseph Akuze, Ane B. Fisker, Justiniano S. D. Martins, Amabelia Rodrigues, Sanne M. Thysen, Gashaw Andargie Biks, Solomon Mokonnen Abebe, Tadesse Awoke Ayele, Telake Azale Bisetegn, Tadess Guadu Delele, Kassahun Alemu Gelaye, Bisrat Misganaw Geremew, Lemma Derseh Gezie, Tesfahun Melese, Mezgebu Yitayal Mengistu, Adane Kebede Tesega, Temesgen Azemeraw Yitayew, Simon Kasasa, Edward Galiwango, Collins Gyezaho, Judith Kaija, Dan Kajungu, Tryphena Nareeba, Davis Natukwatsa, Valerie Tusubira, Yeetey A. K. Enuameh, Kwaku P. Asante, Francis Dzabeng, Seeba Amenga Etego, Alexander A. Manu, Grace Manu, Obed Ernest Nettey, Sam K. Newton, Seth Owusu-Agyei, Charlotte Tawiah, Charles Zandoh, Nurul Alam, Nafisa Delwar, M. Moinuddin Haider, Ali Imam, Kaiser Mahmu, Angela Baschieri, Simon Cousens, Vladimir S. Gordeev, Victoria Ponce Hardy, Doris Kwesiga, Kazuyo Machiyama

**Affiliations:** 1 Maternal, Adolescent, Reproductive & Child Health (MARCH) Centre, London School of Hygiene & Tropical Medicine, London, UK; 2 Department of Health Policy, Planning and Management, Makerere University School of Public Health, Kampala, Uganda; 3 Centre of Excellence for Maternal Newborn and Child Health Research, Makerere University, Kampala, Uganda; 4 Health Systems and Population Studies Division, icddr,b, Dhaka, Bangladesh; 5 IgangaMayuge Health and Demographic Surveillance System, Makerere University Centre for Health and Population Research, Makerere, Uganda; 6 Kintampo Health Research Centre, Kintampo, Ghana; 7 Bandim Health Project, Bissau, Guinea-Bissau; 8 Research Centre for Vitamins and Vaccines, Statens Serum Institut, Copenhagen, Denmark; 9 Department of Clinical Research, Open Patient data Explorative Network (OPEN), University of Southern Denmark, Odense, Denmark; 10 Department of Epidemiology and Biostatistics, Institute of Public Health, University of Gondar, Gondar, Ethiopia; 11 Department of Public Health Sciences, Karolinska Institutet, Stockholm, Sweden

**Keywords:** Stillbirth, Household survey, Foetal loss, Demographic and Health Surveys, Low- and middle-income country, Perinatal death, Risk factor, Pregnancy, Maternity care, Determinants

## Abstract

**Background:**

Household surveys remain important sources of maternal and child health data, but until now, standard surveys such as Demographic and Health Surveys (DHS) have not collected information on maternity care for women who have experienced a stillbirth. Thus, nationally representative data are lacking to inform programmes to address the millions of stillbirths which occur annually.

**Methods:**

The EN-INDEPTH population-based survey of women of reproductive age was undertaken in five Health and Demographic Surveillance System sites in Bangladesh, Ethiopia, Ghana, Guinea-Bissau and Uganda (2017–2018). All women answered a full birth history with additional questions on pregnancy losses (FBH+) or full pregnancy history (FPH). A sub-sample, including all women reporting a recent stillbirth or neonatal death, was asked additional maternity care questions. These were evaluated using descriptive measures. Associations between stillbirth and maternal socio-demographic characteristics, babies’ characteristics and maternity care use were assessed using a weighted logistic regression model for women in the FBH+ group.

**Results:**

A total of 15,591 women reporting a birth since 1 January 2012 answered maternity care questions. Completeness was very high (> 99%), with similar proportions of responses for both live and stillbirths. Amongst the 14,991 births in the FBH+ group, poorer wealth status, higher parity, large perceived baby size-at-birth, preterm or post-term birth, birth in a government hospital compared to other locations and vaginal birth were associated with increased risk of stillbirth after adjusting for potential confounding factors. Regarding association with reported postnatal care, women with a stillbirth were more likely to report hospital stays of > 1 day. However, women with a stillbirth were less likely to report having received a postnatal check compared to those with a live birth.

**Conclusions:**

Women who had experienced stillbirth were able to respond to questions about pregnancy and birth, and we found no reason to omit questions to these women in household surveys. Our analysis identified several potentially modifiable factors associated with stillbirth, adding to the evidence-base for policy and action in low- and middle-income contexts. Including these questions in DHS-8 would lead to increased availability of population-level data to inform action to end preventable stillbirths.

**Supplementary Information:**

The online version contains supplementary material available at 10.1186/s12963-020-00240-1.

## Key findings

**Table:** 

**What is new?**
• **What was known already:** Household surveys could potentially provide population-based data on the majority of the >2 million global stillbirths annually. Standard household surveys such as Demographic and Health Surveys (DHS) until now have not collected information on maternity care for women who experienced a stillbirth, and nationally representative data to inform programmes and track progress towards ending preventable stillbirths are lacking. • **What was done:** As part of the EN-INDEPTH population-based survey of 69,176 women of reproductive age in five countries, we evaluated the use of DHS-7 questions on maternity care for women with a recent (last 5 years) live or stillbirth and assessed the association between stillbirth and the woman’s socio-demographic characteristics, maternity care utilisation and the baby’s characteristics.
**What was found?**
• **Measuring maternity care:** o ***Completeness of responses:*** Questions on maternity care were almost universally answered by women, including those experiencing a stillbirth (> 99.5% responses complete, < 10% ‘don’t know’ responses). o ***Differences in responses for women with live births or stillbirths***: Distribution of responses was similar for live and stillbirths for timing and frequency of antenatal care and timing of postnatal care, but different for length of postnatal care.
• **Measuring factors associated with stillbirth:** o Poorer wealth status, higher parity, large perceived size-at-birth, preterm or post-term birth, birth in a government hospital compared to other locations and vaginal birth were associated with increased risk of stillbirth after adjusting for potential confounding factors.
**What next in measurement and research?**
• **Measurement improvement now:** Household surveys should include questions to women with stillbirths, since answers by these women are complete and may provide useful population level information to inform action to end preventable stillbirths. These questions are now included in DHS-8, with a change made from DHS-7 based on our findings. Our analysis identified several potentially modifiable factors associated with stillbirth, adding to the evidence-base for policy and action in low- and middle-income contexts. • **Research needed:** The use of improved stillbirth outcome measurement (vital status, gestational age and weight) is required to further increase the accuracy and usefulness of stillbirth-related information in household surveys. Investment in routine registers, improved data flow and accountability for the use of stillbirth data (including mortality audit and response) could help accelerate the prevention of millions of stillbirths. Importantly, as around 80% of the world’s births are now in facilities, better care is possible.

## Background

Stillbirth is a major health challenge: approximately 2.6 million stillbirths occurred globally in 2015, 98% of which were in low- and middle-income countries (LMICs) and most of which were preventable [1, 2]. To accelerate change, the Every Newborn Action Plan (ENAP) included a target of < 12 stillbirth per 1000 total births in every country by 2030 [1]. To meet this goal, it is necessary to improve understanding of and address factors associated with stillbirth. However, the lack of population-level data on stillbirth prevalence and associated factors is impeding progress [2–4].

Most data on factors associated with stillbirth come from high-income countries [5]. Known risk factors globally include maternal factors (age; infections [e.g. syphilis, HIV and malaria]; non-communicable disorders [e.g. obesity, diabetes and hypertension]) and foetal factors (e.g. male sex, pregnancy > 42 weeks, premature birth, congenital malformations and rhesus disease) [2, 6, 7] (Additional file 1). Other factors such as birth interval and violence against pregnant women are thought to be important, but data are limited [2]. Many of these factors are also associated with increased maternal and neonatal mortality; thus, addressing these has the potential for a triple return on investment, improving survival and well-being for both the mother and baby, and improving long-term child-development [8].

The majority of LMICs rely on household surveys, notably Demographic and Health Surveys (DHS), for information on stillbirths [9]. These are important sources of population-level information about maternal characteristics and coverage of maternity care for all birth outcomes [9]. By maternity care, we mean care received during pregnancy (notably routine antenatal contact), intrapartum (notably skilled attendance) and postnatally (routine postnatal contact). Coverage and quality of antenatal and delivery care can have a substantial impact on pregnancy outcomes, and thus, it is important that these are captured for stillbirths to identify where and for whom care needs to be improved.

The standard DHS-7 women’s questionnaire and other large-scale surveys such as the National Family Health Survey (NFHS) only captured data on maternal health service use for pregnancies resulting in a live birth, not pregnancies ending in stillbirth [10–12]. This exclusion may introduce bias, for example, when this data is used to understand health service use or determinants of mortality, as women who experience a loss may have had different levels of health care quality and utilisation. It has also led to a paucity of data to understand country-specific determinants and factors associated with stillbirth in LMICs. A few recent publications have demonstrated that when such data are available, more understanding and insight into stillbirths can be obtained, which can be used to target action to end preventable stillbirths and to improve care for affected women [13–15]. DHS-8 now includes these questions for all births and previous studies have also provided population-level data on stillbirths [15–17]; however, the feasibility of asking these in household surveys across a variety of settings and in-depth analyses on the quality of these data have not yet been reported.

This paper is part of a series from the Every Newborn- International Network for the Demographic Evaluation of Populations and their Health (EN-INDEPTH) study in five Health and Demographic Surveillance System (HDSS) sites in sub-Saharan Africa and Asia. In this study, we evaluate the use of existing DHS-7 questions to capture information regarding pregnancy care for women experiencing a stillbirth in population-based surveys, and also explore factors associated with stillbirth and care in the EN-INDEPTH survey.

This paper has three objectives:

1. ***Evaluate data completeness and responses*** for standard DHS-7 questions on maternity care amongst mothers, comparing those with liveborn and stillborn babies.
2. ***Assess the association between stillbirth and the following factors***: socio-demographic, maternity care utilisation before and during delivery and babies’ characteristics.
3. ***Assess the association between stillbirth and reported postnatal care*** for the woman.

## Methods

### EN-INDEPTH study design and setting

**Fig. 1:**
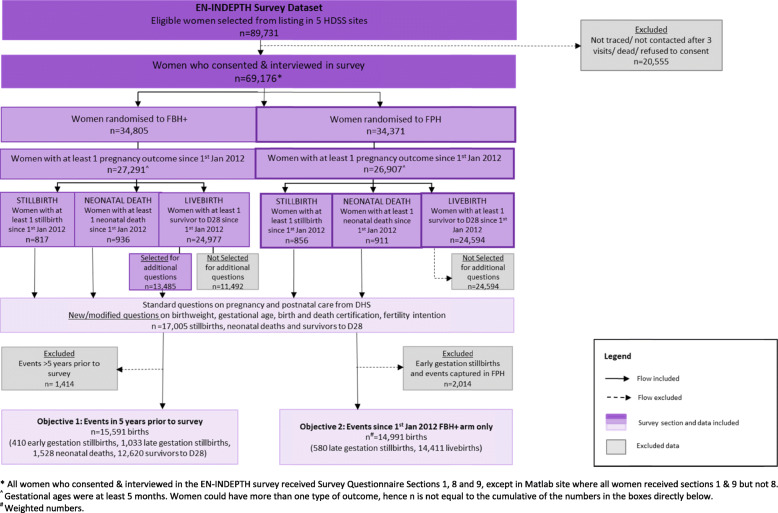
Flow diagram of EN-INDEPTH study population and data included in stillbirth analyses

The EN-INDEPTH study involved a cross-sectional survey conducted between July 2017 and August 2018 of women aged 15–49 years living in five HDSS sites: Bandim in Guinea-Bissau, Dabat in Ethiopia, IgangaMayuge in Uganda, Matlab in Bangladesh and Kintampo in Ghana (Fig. 1; Additional file 2.1). The study protocol and main paper are published elsewhere [18, 19]. The primary objective of the study was to compare two methods of retrospective recording of pregnancy outcomes in surveys: a full birth history with additional questions on pregnancy losses (FBH+) and a full pregnancy history (FPH) [18, 19]. The main paper found that late gestation stillbirth rates (stillbirths at seven or more months of gestation) in the 5 years prior to the survey were 15.2 per 1000 total births in the FBH+ arm and 17.4 per 1000 births in the FPH arm [19]. There was large variation in late gestation stillbirth rates (SBRs) across HDSS sites (SBR 8.1 to 20.2 within the FBH+ arm and 10.6 to 25.5 within the FPH arm). Only a small number of women reported more than one late gestation stillbirth in the last 5 years [19].

For the survey, all women provided information on all live births in their lifetime and pregnancy losses since 1 January 2012 (FBH+), or all pregnancies in their lifetime regardless of the outcome (FPH). A subset of women were asked additional questions about their pregnancies and births: women reporting a stillbirth (including both early gestation stillbirths at 5 or 6 months and late gestation stillbirths at 7 or more months), or a neonatal death (before 28 days of life) since 1 January 2012, and a subset of women with a live birth surviving the neonatal period (further details of this selection are included in Additional file 2.2). Stillbirths were distinguished from neonatal deaths in both the FPH and FBH+ using women’s responses to standard DHS-7 questions (FBH+) or questions on all pregnancies (FPH) (see Additional file 2.3A for details of questions). Where selected women reported more than one of each specific pregnancy outcome, questions were only asked of the most recent event for each outcome.

Interviewers were recruited locally and were familiar with the culture and dialect of the study area. Both woman and interviewer data were collected on Android tablets using the Survey Solutions data collection and management system, which included built-in range checks and error messages for some questions [20]. Following completion of data collection, data from the five HDSS sites were anonymised by local HDSS scientists, encrypted and then shared [18]. Data management and analysis were done using Stata version 15.1.

### Methods by objective

#### Objective 1: Data completeness and responses for DHS-7 questions on maternity care, comparing those with liveborn and stillborn babies

Women selected to respond to the additional questions in the EN-INDEPTH survey were asked a shortened form of the standard DHS-7 core women’s questionnaire. It included pregnancy and postnatal care module for each birth event (Additional file 2.3B). Consistent with DHS, analysis was limited to information provided for events in the 5 years prior to the survey. Information on pregnancy losses was presented separately for late gestation (7 months or more of gestation) and early gestation (5 or 6 months of gestation). It was assumed that whether a woman was interviewed using the FBH+ or FPH would not impact on her responses to the additional questions [19].

Each question was assessed for completeness, ‘don’t know’ responses and response distribution. The completeness and the responses for stillbirths were compared to neonatal deaths and children surviving the neonatal period using descriptive statistics and chi-square tests.

#### Objectives 2 and 3: Associations between stillbirth and women’s socio-demographic characteristics, maternity care utilisation and babies’ characteristics

We analysed the association between stillbirth and potentially related socio-demographic, baby and maternity care factors based on previous LMIC studies and availability in the survey dataset (Additional file 1). The analysis was restricted to women in the FBH+ arm, as women with surviving children in the FPH were not asked the additional questions. Sample weights were applied in all analyses using the *svyset* command to account for the different probability of a neonatal death being included, compared to a live birth surviving the neonatal period, given that women’s responses may vary for these two groups (Additional file 2.4). Only late gestation stillbirths (at 7 months’ gestation or more) were included in the main analyses.

The overall and stratum-specific stillbirth numbers and rates were calculated. Variables with more than 5% of values missing and those without strong evidence of association with stillbirths (*p* > 0.1) on bivariate analysis were excluded from further analysis. Logistic regression models were constructed using a hierarchical approach [21,22] including first background factors, then antenatal factors, then delivery factors and finally baby factors and were used to quantify associations between these factors and stillbirth (Additional file 1).

Separate logistic regression models were constructed to assess the association of stillbirth with reported postnatal care for the woman.

Results are reported in accordance with STROBE Statement checklists for cross-sectional studies [23] (Additional file 3).

## Results

Overall 69,176 women completed the EN-INDEPTH survey. The use of DHS-7 questions on pregnancy and postnatal care were evaluated for 15,591 births, including early gestation stillbirths, which took place in the 5 years prior to the survey (Fig. 1). Further analysis was undertaken for 14,991 live births and late gestation stillbirths since 1 January 2012, which were captured in the FBH+ arm (Fig. 1). This analysis explored the associations between stillbirth and other factors relating to maternal socio-demographic factors, maternity care utilisation and the babies’ characteristics.

### Objective 1: Data completeness and responses for DHS-7 questions on maternity care, comparing those with liveborn and stillborn babies

More than 99.5% of data for each of the existing DHS-7 questions on pregnancy and postnatal care were complete for all 15,591 births in the 5 years prior to the survey. The proportion of ‘don’t know’ responses did not vary by the birth outcome and was <10% for all questions asked (Additional file 4.1). Only one question, which asked about timing of postnatal care, did not have built-in range checks, and the proportion of responses of out-of-range was low (0–1.3%) (Additional file 4.2).

Compared to women with children surviving the neonatal period, a higher proportion of women with late gestation stillbirths reported having seen a doctor for ANC (26.9% vs 16.7%; *p* < 0.0001), having a skilled provider as birth attendant (71.3% vs 58.7%; *p* < 0.0001), an emergency caesarean section (10.8% vs 7.1%; *p* < 0.0001) and a hospital stay over 3 days (42.6% vs 27.0%; *p* < 0.0001); a lower proportion reported giving birth at home (25.6% vs 33.7%; *p* < 0.0001) (Table 1, Additional file 4.3). Reported proportions for most antenatal care indicators were similar between women with late gestation stillbirths and neonatal deaths including provider type, number of visits, place and timing of first visit. However, there is some evidence that women with a late gestation stillbirth were more likely to deliver in a health facility (*p* = 0.046) with a skilled provider (*p* = 0.085) compared to women with a neonatal death, and that women with a late gestation stillbirth were more likely to have a hospital stay of three or more days (*p* = 0.009) (Table 1, Additional file 4.3).

**Table 1:** Reported maternity care for births in 5 years preceding EN-INDEPTH survey by outcome (*n* = 15,591)

	Children surviving the neonatal period *n* = 12,620		Neonatal death *n* = 1528		Late gestation stillbirth *n* = 1033		Early gestation stillbirth *n* = 410	
	*n*	%	*n*	%	*n*	%	*n*	%
**Antenatal care provider**^**a**^								
Doctor	2106	16.7	379	24.8	278	26.9	65	15.9
Midwife/nurse	8329	66.0	938	61.4	640	62.0	224	54.6
Other	1053	8.3	72	4.7	30	2.9	16	3.9
No ANC	1131	9.0	138	9.0	80	7.7	103	25.1
**Number of ANC visits**								
0	1131	9.0	138	9.0	80	7.7	103	25.1
1	489	3.9	97	6.4	66	6.4	63	15.4
2	887	7.0	135	8.8	96	9.3	70	17.1
3	1920	15.2	212	13.9	167	16.2	60	14.6
4 or more	7263	57.6	827	54.1	531	51.4	90	22.0
Not known	925	7.3	117	7.7	88	8.5	22	5.4
**Timing of 1st ANC visit**^**b**^								
First trimester	1725	15.0	255	18.4	171	18.0	88	28.9
Second trimester	8746	76.1	1005	72.4	685	72.3	209	68.5
Third trimester	853	7.4	92	6.6	70	7.4	2	0.7
Not known	164	1.4	37	2.7	22	2.3	6	2.0
**Place of ANC**								
Government facility only	7481	59.3	846	55.4	544	52.7	191	46.6
Private facility only	2478	19.6	390	25.5	266	25.8	71	17.3
Government and private	473	3.8	51	3.3	56	5.4	7	1.7
Other only	1056	8.4	102	6.7	82	7.9	36	8.8
**Birth attendant**^**c**^								
Skilled provider	7411	58.7	1041	68.1	736	71.3	258	62.9
Unskilled provider	4929	39.1	447	29.3	250	24.2	71	17.3
None	278	2.2	38	2.5	41	4.0	78	19.0
**Place of birth**								
A home	4252	33.7	446	29.2	264	25.6	143	34.9
Government hospital	2854	22.6	430	28.1	421	40.8	136	33.2
Government health centre	2365	18.7	243	15.9	87	8.4	29	7.1
Private sector	2092	16.6	272	17.8	184	17.8	62	15.1
Other	1055	8.4	136	8.9	71	6.9	37	9.0
**Mode of delivery**								
Elective caesarean section	805	6.4	90	5.9	48	4.7	8	2.0
Emergency caesarean section	894	7.1	160	10.5	112	10.8	15	3.7
Vaginal birth	10,919	86.5	1277	83.6	867	83.9	384	93.7
**Length of stay at facility**								
0 to < 6 h	1259	16.7	127	12.8	81	11.7	38	16.7
6 to < 12 h	1301	17.3	94	9.5	58	8.4	25	11.0
12 to < 24 h	779	10.3	89	9.0	43	6.2	19	8.3
1–2 days	2165	28.7	321	32.4	216	31.2	101	44.3
3 or more days	2036	27.0	360	36.3	295	42.6	45	19.7
**Postnatal check**								
Yes	6498	85.4	847	84.5	596	84.3	184	79.0
No	1109	14.6	155	15.5	111	15.7	49	21.0

Reported maternity care for all births asked additional questions on pregnancy and birth in the EN-INDEPTH survey

^a^Refers to the highest level provider. ANC provider not considered in further analyses in view of known concerns regarding the accuracy of women’s report of this

^b^For categorisation, trimesters were defined as first (< 13 weeks, < 3 months), second (13–27 weeks, 3–6 months), third (28 weeks, 7 months onwards)

^c^In view of potential difficulty of women knowing the skill of her provider, ‘skilled and unskilled’ healthcare provider groups are merged

**Fig. 2:**
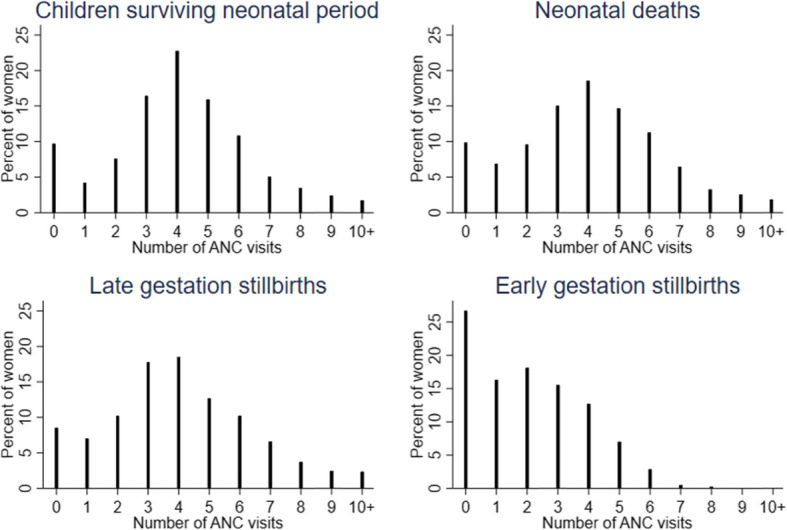
Distribution of number of antenatal care visits by outcome (*n* = 15,591)

Around 95% of women, regardless of birth outcome, reported age at first antenatal care (ANC) in months rather than weeks. The distribution of gestation at first ANC was similar for stillbirths compared to live births (Additional file 4.4). The number of ANC visits was similar for late gestation stillbirths and live births, but fewer for early gestation stillbirths (Fig. 2). Responses regarding length of stay at facility after delivery showed a plausible distribution, with a higher proportion of women with a late gestation stillbirth or neonatal death reporting staying more than 24 h, compared to women whose children survived the neonatal period (Additional file 4.5). The distribution of responses regarding the time from delivery to first postnatal care visit was similar across outcomes (Additional file 4.6).

### Objective 2: Associations between stillbirth and woman’s socio-demographic, maternity care utilisation and baby’s characteristics

Excluding early gestation stillbirths, there were 14,991 total births and 227 late gestation stillbirths (weighted) since 1 January 2012 amongst women in FBH+ arm, giving an overall stillbirth rate of 15.2 per 1000 total births (95%CI 14.0–16.5) (Table 2). Stillbirth rates varied by site, woman’s age, parity, length of gestation, number of ANC visits, birthplace, birth attendant type and mode of delivery (*p* < 0.05) (Tables 2 and 3).

**Table 2:** Women and baby characteristics by late gestation stillbirth rates (FBH+ only, *n* = 14,991)

	No. of births (% of total births^**a**^)		No. of stillbirths	Stillbirth rate per 1000 total births
	*n*	%	*n*	*n* (95%CI)
**Overall**	14,991	100	227	15.2 (14.0–16.5)
**Site**				
Bandim	2713	18.1	54	20.0 (16.9–23.7)
Dabat	2020	13.5	18	9.0 (6.7–12.1)
IgangaMayuge	1976	13.2	16	8.1 (5.8–11.1)
Matlab	4657	31.1	79	17.0 (14.8–19.6)
Kintampo	3626	24.2	60	16.5 (12.7–21.2)
**Age-group (years)**^**b**^				
15–24	3743	25.0	47	12.5 (10.4–14.9)
25–29	4189	28.0	59	14.1 (12.1–16.6)
30–34	3318	22.1	56	16.9 (14.3–20.1)
35–39	2350	15.7	38	16.2 (13.2–19.8)
40–50	1389	9.3	27	19.7 (15.5–25.0)
**Education-status**				
No education	4112	27.4	62	15.1 (12.9–17.8)
Primary only	4674	31.2	73	15.5 (13.4–18.0)
Primary and secondary	5149	34.4	82	15.9 (13.9–18.2)
Higher	1056	7.0	11	10.1 (6.9–14.8)
**Wealth quintile**				
Poorest	3290	22.0	55	16.9 (14.3–19.9)
Poorer	3004	20.0	51	16.9 (14.2–20.2)
Middle	2909	19.4	42	14.4 (11.8–17.4)
Wealthier	2959	19.7	46	15.4 (12.8–18.5)
Wealthiest	2829	18.9	34	11.9 (9.6–14.9)
**Parity**				
1	2504	16.7	17	7.0 (5.2–9.3)
2	3802	25.4	49	12.8 (10.7–15.1)
3	2868	19.1	53	18.5 (15.6–21.9)
4	1874	12.5	37	19.6 (15.9–24.3)
5+	3943	26.3	71	18.1 (15.6–210)
**Gestation (months)**				
≤ 6	26	0.2	-----	-----
7	218	1.5	44	202.5 (165.0–245.9)
8	884	5.9	46	52.0 (42.8–63.0)
9	12,598	84.2	121	9.6 (8.6–10.7)
≥ 10	1231	8.2	17	13.6 (10.0–18.4)
**Perceived size at birth**				
Very large	1129	7.6	17	15.5 (11.5–20.8)
Larger than average	2458	16.5	49	19.9 (16.6–23.9)
Average	9082	61.0	92	10.1 (8.9–11.5)
Smaller than average	1567	10.5	20	12.6 (9.5–16.7)
Very small	611	4.4	16	26.1 (19.0–35.8)

^a^Weighted number

^b^Woman’s age at survey

**Table 3:** Maternity care characteristics by late gestation stillbirth rates (FBH+ only, *n* = 14,991)

	No. of births (% of total births^**a**^)		No. of stillbirths	Stillbirth rate per 1000 total births
	*n*	%	*n*	*n* (95%CI)
**Overall**	14,991	100	227	15.2 (14.0–16.5)
**Number of ANC visits**				
0	1073	7.2	21	19.8 (15.0–26.1)
1	709	4.7	15	20.9 (15.0–29.0)
2	1110	7.3	21	18.6 (14.1–24.5)
3	2119	14.1	36	16.8 (13.7–20.7)
4 or more	8867	59.2	113	12.8 (11.3–14.4)
Not known	1117	7.5	20	18.0 (13.6–23.9)
**Timing of 1st ANC visit**				
First trimester	2140	15.4	35	16.3 (13.2–20.1)
Second trimester	10,405	74.8	150	14.4 (13.0–15.9)
Third trimester	1099	7.9	16	14.2 (10.3–19.4)
Not known	272	2.0	4	15.4 (8.0–29.4)
**Place of ANC**				
Government facility only	7920	56.9	113	14.2 (12.6–16.0)
Private facility only	3686	26.5	58	15.7 (13.3–18.4)
Government and private	583	4.2	11	19.5 (13.2–28.7)
Other only	1726	12.4	22	13.0 (10.0–16.8)
**Birth attendant**^**b**^				
Skilled provider	9671	64.5	158	16.3 (14.8–18.1)
Unskilled provider	4953	33.1	60	12.1 (10.3–14.2)
None	361	2.4	8	21.1 (13.4–32.9)
**Place of birth**				
A home	4718	31.5	60	12.8 (10.9–15.0)
Government hospital	3032	20.2	86	28.3 (24.6–32.5)
Government health centre	2544	17.0	19	7.3 (5.5–9.7)
Private sector	3011	20.1	41	13.5 (11.1–16.4)
Other	1678	11.2	20	12.0 (9.0–15.9)
**Mode of delivery**				
Elective caesarean section	1283	8.6	9	7.4 (4.9–11.1)
Emergency caesarean section	1214	8.1	24	20.0 (15.5–25.7)
Vaginal birth	12,487	83.3	192	15.4 (14.0–16.8)
**Length of stay at facility**^**c**^				
0 to < 6 h	1203	13.3	19	16.1 (12.1–21.4)
6 to < 12 h	1284	14.2	12	9.2 (6.4–13.1)
12 to < 24 h	857	9.5	8	9.7 (6.4–14.8)
1–2 days	2658	29.5	46	17.3 (14.3–20.9)
3 or more days	3015	33.4	60	20.0 (17.0–23.6)
**Postnatal check**				
Yes	7999	87.7	125	15.6 (13.9–17.5)
No	1122	12.3	23	20.6 (15.9–26.8)

^a^Weighted number

^b^In view of potential difficulty of women knowing the skill of her provider, ‘skilled and unskilled’ healthcare provider groups are merged

^c^Facility births only

**Fig. 3:**
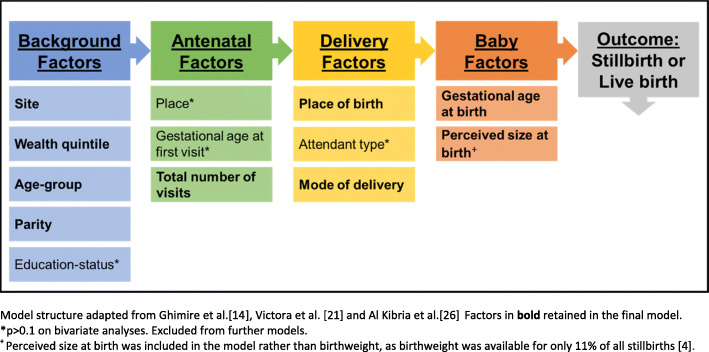
Modelling framework for factors associated with stillbirth, EN-INDEPTH study

Four levels of factors associated with stillbirth events were considered in a hierarchical model (Fig. 3). Five variables were excluded from the multivariable analysis. Maternal education, ANC place, gestational age at first ANC visit and type of birth attendant had *p* > 0.1 on bivariate analysis. Birthweight was excluded as data were only available for 11% of stillbirths [24].

After adjusting for other variables in the multivariable model, we found poorer wealth quintile, higher parity, preterm or post-term birth, large perceived size-at-birth, birth in a government hospital compared to home and vaginal birth compared to caesarean were associated with increased stillbirth risk (Table 4).

**Table 4:** Multivariable analysis of association between stillbirth and woman’s and baby’s and maternity care characteristics (FBH+ only, *n* = 14,991)

	Crude odds ratio (95% CI)	***p*** value (Wald test)	Adjusted odds ratio (95% CI)	***p*** value (Wald test)
**Site**				
Bandim	1.17 (0.94–1.47)	< 0.001	1.17 (0.77–1.77)	< 0.001
Dabat	0.53 (0.38–0.73)		0.38 (0.22–0.66)	
IgangaMayuge	0.47 (0.33–0.66)		0.19 (0.11–0.33)	
Matlab	1.00 (base)		1.00 (base)	
Kintampo	0.96 (0.77–1.20)		0.34 (0.21–0.54)	
**Age-group (years)**^**a**^				
15–24	1.00 (base)	0.020	1.00 (base)	0.206
25–29	1.14 (0.89–1.45)		0.77 (0.55–1.07)	
30–34	1.36 (1.06–1.75)		0.65 (0.44–0.95)	
35–39	1.30 (0.99–1.71)		0.61 (0.40–0.95)	
40–50	1.59 (1.17–2.16)		0.69 (0.42–1.12)	
**Education-status**				
No education	1.00 (base)	0.173	-----	-----
Primary only	1.03 (0.82–1.28)		-----	
Primary and secondary	1.05 (0.85–1.31)		-----	
Higher	0.66 (0.43–1.01)		-----	
**Wealth quintile**				
Poorest	1.00 (base)	0.096	1.00 (base)	0.015
Poorer	1.01 (0.79–1.29)		0.92 (0.68–1.24)	
Middle	0.85 (0.66–1.10)		0.75 (0.55–1.02)	
Wealthier	0.91 (0.71–1.17)		0.75 (0.55–1.02)	
Wealthiest	0.71 (0.53–0.93)		0.57 (0.40–0.80)	
**Parity**				
1	1.00 (base)	< 0.001	1.00 (base)	< 0.001
2	1.84 (1.31–2.59)		2.23 (1.45–3.42)	
3	2.69 (1.92–3.77)		3.78 (2.36–6.06)	
4	2.85 (1.98–4.10)		5.44 (3.15–9.38)	
5+	2.62 (1.89–3.65)		6.57 (3.74–11.54)	
**Gestational age at birth (months)**				
7	26.2 (19.91–34.56)	< 0.001	36.0 (24.56–52.83)	< 0.001
8	5.67 (4.48–7.16)		8.84 (5.92–13.22)	
9	1.00 (base)		1.00 (base)	
≥ 10	1.42 (1.02–1.98)		1.51 (1.05–2.19)	
**Perceived size at birth**				
Very large	1.54 (1.10–2.14)	< 0.001	1.83 (1.23–2.72)	< 0.001
Larger than average	2.00 (1.58–2.50)		2.34 (1.78–3.07)	
Average	1.00 (Base)		1.00 (base)	
Smaller than average	1.25 (0.91–1.71)		0.69 (0.47–1.01)	
Very small	2.62 (1.84–3.72)		0.58 (0.35–0.96)	
**Number of ANC visits**				
0	1.00 (base)	< 0.001	1.00 (base)	0.170
1–3	0.91 (0.66–1.25)		0.96 (0.63–1.48)	
4 or more	0.64 (0.47–0.87)		0.73 (0.47–1.14)	
Not known	0.91 (0.61–1.36)		0.75 (0.42–1.35)	
**Timing of 1st ANC visit**				
First trimester	1.00 (base)	0.754	-----	-----
Second trimester	0.88 (0.69–1.12)		-----	
Third trimester	0.87 (0.58–1.26)		-----	
Not known	0.94 (0.47–1.89)		-----	
**Place of ANC**				
Government facility only	1.00 (base)	0.284	-----	-----
Private facility only	1.10 (0.90–1.35)		-----	
Government and private	1.38 (0.91–2.08)		-----	
Other only	0.91 (0.68–1.22)		-----	
**Birth attendant**				
Healthcare provider^b^	1.00 (Base)	0.139	-----	-----
None	1.42 (0.89–2.26)		-----	
**Place of birth**				
A home	1.00 (base)	< 0.001	1.00 (base)	< 0.001
Government hospital	2.25 (1.81–2.79)		3.96 (2.77–5.65)	
Government health centre	0.57 (0.41–0.79)		0.73 (0.46–1.13)	
Private sector	1.06 (0.82–1.36)		1.49 (1.01–2.20)	
Other	0.94 (0.67–1.30)		0.80 (0.54–1.19)	
**Mode of delivery**				
Elective caesarean section	0.48 (0.31–0.73)	< 0.001	0.19 (0.11–0.34)	< 0.001
Emergency caesarean section	1.31 (1.00–1.72)		0.55 (0.34–0.87)	
Vaginal birth	1.00 (base)		1.00 (base)	

Adjusted for site, age group, wealth quintile, parity, gestation, perceived size at birth, number of ANC visits, place of birth and mode of delivery

^a^Woman’s age at survey

^b^In view of potential difficulty of women knowing the skill of a given provider, ‘skilled and unskilled’ healthcare provider groups have been merged

#### Associations relating to women’s socio-demographic and baby’s characteristics after adjustment

Women in the wealthiest quintile had 43% lower odds of reporting a stillbirth compared to women in the poorest quintile (aOR 0.57 (95% CI 0.40–0.80)). For parity, the odds of reporting a stillbirth increased as parity increased with adjusted odds ratios ranging from 2.23 (95% CI 1.45–3.42) for women with two children to 6.57 (95% CI 3.74–11.54) for those with 5+ children, compared to women with one child only. Both preterm and post-term births were associated with increased odds of reporting a stillbirth when compared with term births, with effect sizes higher for preterm births: aOR 36.0 (95% CI 24.56–52.83) for 7-month gestation; aOR 8.84 (95% CI 5.92–13.22) for 8-month gestation; and aOR 1.51 (95% CI 1.05–2.19) for ≥ 10-month gestation. For perceived size at birth, women with ‘very large’ and ‘larger than average’ babies were 1.83 times (95% CI 1.23–2.72) and 2.34 times (95% CI 1.78–3.07) more likely to report a stillbirth respectively; however, women with ‘very small’ babies were 42% (aOR 0.58 (0.35–0.96)) less likely (Table 4).

#### Associations relating to maternal care characteristics after adjustment

Women who gave birth in a government hospital had increased odds of reporting a stillbirth (aOR 3.96 (95% CI 2.77–5.65)) compared to women who delivered at home. Other birth locations were not significantly associated with the risk of stillbirths, except private sector births compared to home births (aOR 1.49 (95% CI 1.01–2.20)). For mode of delivery, women who had elective and emergency caesarean section were less likely to report a stillbirth compared to women who had vaginal births: aOR 0.19 (95% CI 0.11–0.34) and aOR 0.55 (95% CI 0.34–0.87) respectively (Table 4).

### Objective 3: Association between stillbirth and reported postnatal care for the woman

Women with a stillbirth were more likely to report hospital stays of more than one day (1–2 days: aOR 1.93 (95%CI 1.27–2.94); 3 or more days: aOR 3.33 (95%CI 2.02–5.48)) (Table 5). However, despite their higher medical and psychological needs associated with stillbirth, women with a stillbirth outcome were less likely to report having received a postnatal check compared to women with live births (aOR 1.63 (95%CI 1.08–2.45)).

**Table 5:** Multivariable analysis of association between stillbirth and reported care after delivery (FBH+ only, *n* = 14,991)

	Crude odds ratio (95% CI)	***p*** value (Wald test)	Adjusted odds ratio (95% CI)	***p*** value (Wald test)
**Length of stay at facility**				
0 to < 6 h	1.72 (1.15–2.57)	0.0001	1.83 (1.10–3.06)	< 0.001
6 to < 24 h	1.00 (base)		1.00 (base)	
1–2 days	1.85 (1.33–2.59)		1.93 (1.27–2.94)	
3 or more days	2.15 (1.56–2.97)		3.33 (2.02–5.48)	
**Postnatal check**				
Yes	1.00 (base)	0.056	1.00 (base)	< 0.001
No	1.34 (0.99–1.78)		1.63 (1.08–2.45)	

Adjusted for site, age group, wealth quintile, parity, gestation, perceived size at birth, number of ANC visits, place of birth, mode of delivery, length of stay and postnatal check. Consistent with standard DHS questions on postnatal care, these questions were asked for facility births only

## Discussion

We found that women reporting a stillbirth were able to report on the pregnancy and postnatal care they received for pregnancies as often as women whose babies survived the neonatal period, with no difference in completeness or ‘don’t know’ responses. The responses that women gave were consistent with expected patterns. For example, women with early gestation stillbirths reported fewer ANC visits, consistent with their pregnancies lasting only 5 or 6 months, giving less time for routine ANC appointments, even if complications were detected. Women with late gestation stillbirths were less likely to give birth at home and more likely to report having seen a doctor for ANC, having a skilled provider, having an emergency caesarean section and having a hospital stay over 3 days compared to women whose babies survived the neonatal period. These findings are consistent with increased care requirements associated with pregnancy complications.

Lower socioeconomic status was associated with increased risk of stillbirth, and this is consistent with previous reports [2, 25], including a study which found a dose response of increasing intrapartum stillbirth risks as levels of socioeconomic deprivation rose [26]. This might be explained by a complex interplay of factors including poorer access to quality and timely care during pregnancy and delivery. Whilst we found an increased risk of stillbirth with increasing maternal age on crude analysis, this association disappeared after adjusting for other variables in the model, including parity. Surprisingly, we found that reported stillbirth rates were lowest for women for which the index birth was their first birth, with stillbirth risk increasing with parity. This is in contrast to most other studies for which the stillbirth risk is highest for primigravidae. Previous studies have found many barriers to reporting stillbirths in household surveys [27, 28], and the observed decreased risk for first pregnancies may be due to lower reporting. It is plausible that women may be less likely to disclose a pregnancy that ended in a stillbirth if it was her first pregnancy, and she had no subsequent living children. Further research is required to investigate this.

Caesarean section was associated with lower risk of stillbirth. Whilst birth via caesarean section may be medically indicated or the woman’s preference in a minority of pregnancies with a confirmed fetal death, it would be expected that the majority of babies who die in-utero would be delivered vaginally to minimise risks associated with caesarean section for the woman. In the EN-INDEPTH study, overall 5.7% of early gestation and 15.5% of late gestation stillbirths were reported to have been born by caesarean section. If correct, some potential explanations for these relatively high caesarean rates for stillbirths may be failure to detect fetal death in-utero, time-lags between decision for caesarean section in the case of fetal distress and surgery, or cases of diagnosed fetal death where clinicians may opt for caesarean section due to lack of skills or experience in instrumental deliveries, or where there is a need for symphysiotomy and/or destructive delivery.

Very small, larger than average and very large reported baby sizes were associated with increased risks of stillbirth in crude analyses, consistent with previously reported J-shaped risks for mortality by birthweight [29, 30]. We were unable to use recorded or recalled actual birthweight for this analysis due to a large proportion of missing data, with birthweight data available on just 11% of all stillbirths [24]. Whilst maternal perception of size for liveborn babies has been found to be a useful measure at a population level, its reliability in classifying the size of an individual baby is limited [31]. Whilst the majority (99%) of mothers of both live and stillborn babies responded to the ‘size at birth’ question, no research has been undertaken on maternal perceptions of size for non-live births and this information may be even less reliable for stillbirths, as few women in most LMIC settings get to see or hold their stillborn baby [32, 33]. However, larger size would be consistent with increased rates of cephalo-pelvic disproportion and obstructed labour which are common antecedents of term intrapartum stillbirth [2]. The smaller effect for the ‘very large’ compared to ‘larger than average’ may be associated with largest size being associated with healthy babies, and not stillbirths. The strong association of small reported size and stillbirth is not seen after adjusting for other factors, including length of gestation.

After adjusting for other factors in the model, preterm and post-term gestations were both associated with increased stillbirth risks. These findings are consistent with other studies. Post-term gestation is a known risk factor for stillbirth, and most settings with reliable gestational age assessment during pregnancy have introduced policies to induce pregnancies continuing beyond 41 weeks to reduce stillbirth risk [34]. Existing studies show that the proportion of stillbirths over all births at any given gestation decreases with increasing gestational age up to term gestation, with the highest stillbirth risk for the most preterm [35]. Despite the challenges of gestational age assessment in household surveys, our findings also show highest risk at 7 months’ reported gestation [35]. The very high risk reported for births at 7 months’ gestation may also in part be due to higher levels of misclassification of neonatal deaths as stillbirths at these lowest gestations [36, 37].

With more than three-quarters of stillbirths reported to have taken place in facilities, increasing resources are needed to ensure that every woman gets the tailored care that she needs after stillbirth. Women with a stillbirth were more likely to report hospital stays of more than 1 day, which is consistent with the association of stillbirth and increased levels of maternal morbidity. However, having had a stillbirth was also associated with short stays of < 6 h. This finding, if true, is potentially concerning and may indicate a gap in the provision of care for women after stillbirth who are likely to have higher medical and psychological needs compared to women with live births [8]. In addition, after adjusting for other factors, women with a stillbirth outcome were less likely to report having received a postnatal check compared to women with live birth outcomes. This may indicate a perceived or real treatment gap, as these women are unlikely to be receiving the full supportive post-stillbirth care package that they need [38, 39].

Consistent with previous studies [13, 40], we found a higher risk of stillbirth amongst births in government health facilities compared to other facility types or home births. This is likely to represent bias due to different case mixes across the settings, with women at the lowest risk of stillbirth delivering at home or in first level facilities, compared to women with complications detected antenatally or developing during labour who may be transferred to government hospitals. Likewise, women are frequently transferred out of the private sector to government facilities when complications arise, or when fetal death is diagnosed. Reducing stillbirths in government facilities will not only require improvements in the quality of care delivered at the facility, but also strengthening the referral pathways from lower level or private facilities to ensure that women and their babies receive timely emergency interventions if required [41, 42].

Our findings have several implications for policy makers. As a first step, data on women who experience stillbirths need to be collected and documented for evidence-based decision making. Our study shows that collecting such data on women with a stillbirth is feasible now in population-based surveys. However, this should be coupled with strengthening of routine data systems and maternity and perinatal audits to provide more regular updates to improve tracking. As several of the factors associated with stillbirths in our study are potentially modifiable, greater efforts are needed in addressing structural and socio-economic drivers of stillbirths. In addition, women who experience stillbirths need more specific targeting to ensure that they receive quality postnatal care.

### Strengths and limitations

Strengths of this study include the large survey dataset from five LMICs, using standard DHS-7 questions and consistent methods of data collection and analyses. Since our study was undertaken with women in HDSS sites who were under regular surveillance, we acknowledge that their knowledge about stillbirth may differ from women not under surveillance. The data are based on a cross-sectional survey, so there is the potential for recall and social desirability biases. Recall bias could have resulted in the under- or over-reporting of some of the variables. Social desirability bias is particularly relevant in the case of stillbirth due to the highly stigmatised nature of this birth outcome [8, 32, 38, 43, 44], but based on the present data, we are not able to assess the impact on the responses. We found substantial inter-site heterogeneity in stillbirth rates which may in part be due to variation in omission and misclassification of stillbirths across these sites [36]. As highlighted elsewhere, differences in training and translation of the standard study interviewer manual may account for some of these differences [19]. Further work is needed to reduce omission and misclassification of stillbirth events to improve the utility of these data [37].

Whilst we collected information on a range of factors potentially associated with stillbirth, no data were available on some important modifiable factors such as maternal BMI, smoking status, obstetric history, pregnancy symptoms and quality of maternal care [2, 5, 7]. Some of this information could have been captured by the DHS Supplementary Maternal Health Questionnaire, and future research using this module could be continued to further increase utility of information collected, taking into account any additional time implications for data collection [45]. Similar to standard DHS surveys, we only collected birth outcome data from living women; thus, our approach did not capture pregnancies ending in a maternal death, which have a higher risk of stillbirth.

## Conclusions

If the ENAP target of <12 stillbirths per 1000 total births in all countries is to be met by 2030, the current annual rate of 2% reduction must increase more than two-fold [2, 46]. To achieve this, we must act quickly to acquire reliable data to understand factors associated with stillbirth and to monitor progress with prevention measures.

Our study found that women who had experienced stillbirth were able to adequately respond to most questions about maternity care. Our analysis identified several potentially modifiable factors associated with stillbirth, adding to the evidence-base. DHS-8 will now include these questions for women with a stillbirth. This represents an opportunity to provide comparable, nationally representative stillbirth data on a large scale. This information must be analysed and used to provide country-specific data for action by researchers, healthcare providers and policy makers in LMICs. Such progress would help to end preventable stillbirths and improve care for women and families affected by such losses.

## Supplementary Information

**Additional file 1.** Framework for factors associated with stillbirths

**Additional file 2.** Additional methods. Additional file 2.1: Background overview of the five HDSS sites. Additional file 2.2: Details of selection of women with a livebirth surviving the neonatal period. Additional file 2.3: Questions on stillbirths and maternity care asked in EN-INDEPTH survey. Additional file 2.3A: Questions on stillbirths asked in EN-INDEPTH survey1. Additional file 2.3B: Questions on maternity care asked in EN-INDEPTH survey. Additional file 2.4: Calculation of survey weights.

**Additional file 3.** STROBE guidelines checklist

**Additional file 4.** Additional results. Additional file 4.1: Don’t know and missing responses by pregnancy outcomes for selected DHS-7 standard pregnancy and postnatal care questions. Additional file 4.2: Data errors in timing of postnatal care questions. Additional file 4.3: Comparison of selected maternal care indicators by outcome. Additional file 4.4: Distribution of age at first antenatal care visit by outcome. Additional file 4.4A: Distribution of age at first ANC reported in weeks by outcome (n=501). Additional file 4.4B: Distribution of age at first ANC reported in months by outcome (n=13,400). Additional file 4.5: Distribution of post-delivery length of stay in facility in days. Additional file 4.6: Distribution of age at first postnatal care visit in hours

**Additional file 5.** Ethical approval of local Institutional Review Boards

## Data Availability

Data sharing and transfer agreements were jointly developed and signed by all collaborating partners. The datasets generated during the current study are deposited online at 10.17037/DATA.00001556 with data access subject to approval by collaborating parties.

## Abbreviations

ANC: Antenatal care
DHS: Demographic and Health Surveys
ENAP: Every Newborn Action Plan
EN-INDEPTH: Every Newborn-International Network for the Demographic Evaluation of Populations and their Health
FBH+: Full birth history (+ denotes additional questions on pregnancy losses)
FPH: Full pregnancy history
HDSS: Health and Demographic Surveillance System
LMICs: Low- and middle-income countries
SBR(s): Stillbirth rate(s)
