# Supplementary material for: Stillbirth maternity care measurement and associated factors in population-based surveys: EN-INDEPTH study
Source: Popul Health Metr. 2021 Feb 8;19(Suppl 1):11. doi: 10.1186/s12963-020-00240-1 (PMC7869205; doi:10.1186/s12963-020-00240-1)
Supplement: Supplementary file 1 — Additional file 1. Framework for factors associated with stillbirths [file 12963_2020_240_MOESM1_ESM.docx]

**Additional file 1: Framework^1^ for factors associated with stillbirths**


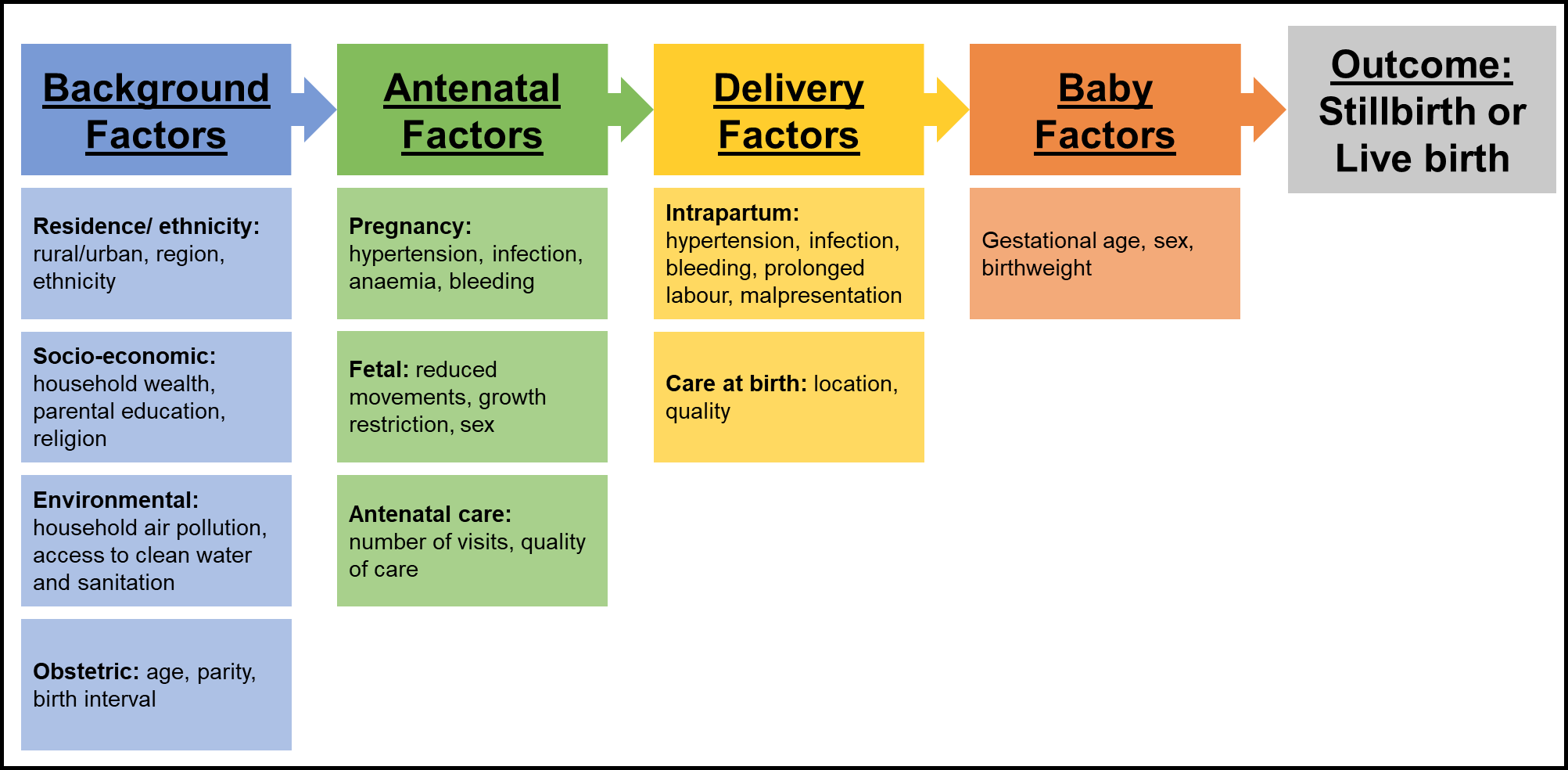


^1^ Model structure adapted from Ghimire et al.[1], Al Kibria et al.[2] and Victora et al.[3]

**References**

1. Ghimire PR, Agho KE, Renzaho AMN, Nisha MK, Dibley M, Raynes-Greenow C: **Factors associated with perinatal mortality in Nepal: evidence from Nepal demographic and health survey 2001–2016**. *BMC Pregnancy and Childbirth* 2019, **19**(1):88.

2. Al Kibria GM, Burrowes V, Choudhury A, Sharmeen A, Ghosh S, Mahmud A, Kc A: **Determinants of early neonatal mortality in Afghanistan: an analysis of the Demographic and Health Survey 2015**. *Globalization and Health* 2018, **14**(1):47.

3. Victora CG, Huttly SR, Fuchs SC, Olinto MTA: **The role of conceptual frameworks in epidemiological analysis: A hierarchical approach**. *International Journal of Epidemiology* 1997, **26**(1):224-227.
