# Supplementary material for: Stillbirth maternity care measurement and associated factors in population-based surveys: EN-INDEPTH study
Source: Popul Health Metr. 2021 Feb 8;19(Suppl 1):11. doi: 10.1186/s12963-020-00240-1 (PMC7869205; doi:10.1186/s12963-020-00240-1)
Supplement: Supplementary file 2 — Additional file 2. Additional methods. Additional file 2.1: Background overview of the five HDSS sites. Additional file 2.2: Details of selection of women with a livebirth surviving the neonatal period. Additional file 2.3: Questions on stillbirths and maternity care asked in EN-INDEPTH survey. Additional file 2.3A: Questions on stillbirths asked in EN-INDEPTH survey1. Additional file 2.3B: Questions on maternity care asked in EN-INDEPTH survey. Additional file 2.4: Calculation of survey weights. [file 12963_2020_240_MOESM2_ESM.docx]

# Additional file 2: Additional methods

## Additional file 2.1: Background overview of the five HDSS sites

|  | **Bandim** | **Dabat** | **IgangaMayuge** | **Matlab** | **Kintampo** |
| --- | --- | --- | --- | --- | --- |
| **Site Information** | | | | | |
| Country | Guinea Bissau | Ethiopia | Uganda | Bangladesh | Ghana |
| HDSS start year | 1978 | 1996 | 2004 | 1966 | 1994 |
| Location | Guinea-Bissau, covering rural and urban | Gondor, Amhara regional state, 821 km northwest of Addis Ababa and 75km north | Iganga and Mayuge districts, approximately, 120km east of capital, Kampala along Kenya-Uganda highway | Matlab Upazila, in Chandpur district, 55km southeast of capital, Dhaka | Within the Kintampo North Municipality and the Kintampo South District of the Brong Ahafo (now Bono East) region of Ghana |
| Population | 180,000 | 69,000 | 83,000 | 230,000 | 153,000 |
| Households | 36,000 | 16,000 | 16,000 | 53,000 | 32,000 |
| Total Fertility Rate | 4.3 | 3.8 | 4.3 | 2.6 | 4.1 |
| **Data capture process** | | | | | |
| Frequency of surveillance rounds | Urban: Monthly  Rural: 2/year (more frequent in some regions) | 2/year | 2/year | 6/year | 1/year |
| Frequency of re-census | Urban: Intervals of 2-7 years dependent on funding  Rural: continuous | Every 7 years | Each update round (Twice a year) | 8 years or more | Last census 2003 |
| Methods for pregnancy identification | Each woman as asked about her pregnancy status and a pregnancy ID is given if found pregnant. | Local guides report pregnancies and births within 48 hours after they have been identified. 83% of the informants are female | Has 64 Community based “scouts”  and Village Health Teams. Both male and female scouts are used | The women age 15-49 years old are given a urine test for pregnancy if pregnancy status is unknown. 100% of the enumerators are female | Community key informants are used to notify pregnancies. Each woman is asked about her pregnancy status |
| Facility births | Urban: 65%  Rural: 39% | 17% | 64% | 69% (Intervention area: 87%; Comparison Area: 50%) | 61% |
| Links to facility data | In national hospital, not in rural | Pilot study ongoing | Not currently | Matlab hospital only (17% of births). The hospital is in the intervention area.[1] | No |
| Data collection | Paper-based | Piloting tablet since mid-2018 | Paper-based | Electronic (Galaxy Tablet) | Paper-based |
| **Availability of outcome indicators** | | | | | |
| Stillbirths | Yes | Yes | Yes | Yes | Yes |
| Neonatal deaths | Yes | Yes | Yes | Yes | Yes |
| Birthweight | Yes - Urban only | Yes | Yes | Yes | Yes – Since 2015 |
| Gestation Age (GA) | Yes – At pregnancy registration | Yes | Yes | Yes | Yes |
| Birth certificate | Yes – On a subset around 400 women | No | No | Yes | No |
| Death certificate | Urban: Yes – asked with verbal autopsy | No | Yes – asked with verbal autopsy | Yes | Yes – asked with verbal autopsy |
| Miscarriages/ Abortions | Yes - Miscarriages only | Yes - Abortions differentiated from miscarriages | Yes - Miscarriages only | Yes - Abortions differentiated from miscarriages | Yes - Abortions differentiated from miscarriages |

## Additional file 2.2: Details of selection of women with a livebirth surviving the neonatal period.

Initial piloting of the EN-INDEPTH survey app tool found it time-consuming to administer. In order to reduce the overall interview time (whilst still reaching the target sample size for the main randomised comparison between FBH+ and FPH), only a subset of women who had at least 1 livebirth that survived the neonatal period since 1^st^ January 2012 were selected to receive the pregnancy and birth module in their questionnaire.

Due to the app set up, it was not possible to select women from the FPH arm for this part of the survey; therefore, only women who were in the FBH+ were eligible for selection. Selection was made at random by the app, with the initial intention that a minimum of 30% of all women interviewed in the FBH+ arm who had a livebirth surviving the neonatal period (i.e. that did not result in a neonatal death) since 1^st^ January 2012 would be selected to receive the pregnancy and birth questionnaire module after the roster. Where a woman had more than one surviving livebirth, only the most recent surviving livebirth was eligible.

The procedure for selecting the sample of women with an eligible livebirth was performed differently across the sites. In Dabat, all women interviewed in the FBH+ with an eligible livebirth received the pregnancy and birth module. In Matlab and Bandim, throughout the whole data collection period, all women in the FBH+ with an eligible livebirth were selected at random to receive the pregnancy and birth module or to skip directly to the next section of the survey. Randomisation was undertaken within the app through the generation of a random number from 0 – 1 for each eligible woman at the start of the survey. Women with a random number of <0.5 were allocated to the FBH+ arm, women with a random number of ≥0.5 were allocated to the FBH arm of the study. Amongst those allocated to FBH+ with an eligible livebirth, additional questions were asked to all women with a randomisation number ≤0.15 (i.e. 30% with an eligible livebirth). In IgangaMayuge and Kintampo, for the first 1 – 2 months of data collection, all women interviewed in the FBH+ with an eligible livebirth were selected to receive the pregnancy and birth module; for the rest of the data collection period, only a random selection of 30% of such (those with a randomisation number ≤0.15) were asked these additional questions.

As a result of the variation in the sampling across sites, overall 50% of women in the FBH+ arm with an eligible livebirth received the pregnancy and birth module. The proportion that completed the module is detailed by site, below:

|  | **Number of women in FBH+ arm completing pregnancy and birth module** | **Percentage of eligible women in FBH+ arm completing pregnancy and birth module** | **Percentage contribution to total surviving livebirths by site** |
| --- | --- | --- | --- |
| **Bandim** | 1,316 | 28.3% | 9.8% |
| **Dabat** | 3,357 | 99.4% | 24.9% |
| **IgangaMaguye** | 1,917 | 64.3% | 14.2% |
| **Matlab** | 2,936 | 29.6% | 21.8% |
| **Kintampo** | 3,951 | 66.7% | 29.3% |
| **Total** | 13,477 | 50.2% | 100.0% |

**Additional file 2.3: Questions on stillbirths and maternity care asked in EN-INDEPTH survey**

### **Additional file 2.3A: Questions on stillbirths asked in EN-INDEPTH survey^1^**

| **DHS standard Approach** | **Domain** | **Question** | **Potential responses** |
| --- | --- | --- | --- |
| DHS-7  FBH+ | Vital Status at birth | Have you ever had a pregnancy that miscarried, was aborted, or ended in a stillbirth? | Yes, No |
|  |  | Since January 2012, have you had any other pregnancies that did not result in a livebirth? | Yes, No |
|  | Duration of pregnancy/ gestational age | How many months pregnant were you when that pregnancy ended? | Numeric integer  (Range: 0-11) |
|  | Event in 5 years prior to survey | When did such pregnancy end? | Months: Single select from list or Don’t Know  Years: Numeric integer (Range: 1980-2018) |
| DHS-8  FPH | Vital Status at birth | Was the baby born alive, born dead, or lost before full term? | - Born alive  - Born dead  - Lost before full term |
|  |  | *If responded ‘born dead’ then asked:*  Did that baby cry, move, or breathe when it was born? | Yes, No |
|  | Duration of pregnancy/ gestational age | How many months did this pregnancy last? | Numeric integer  (Range: 0-11) |
|  | Event in 5 years prior to survey | On what day, month and year did this pregnancy end? | Days: Numeric integer (Range: 1-31)  Months: Single select from list or Don’t Know  Years: Numeric integer (Range: 1980-2018) |

^1^ Questions included above for the FBH+ are the exact questions used in the model questionnaire for DHS-7, asked after the full live birth history. Questions included for the FPH are from the Nepal 2016 DHS FPH module, which are similar to the FPH module included in the core woman’s questionnaire in DHS-8**.**

### **Additional file 2.3B: Questions on maternity care asked in EN-INDEPTH survey**

| **Domain** | **Question** | **Potential responses** |
| --- | --- | --- |
| Antenatal care | Did you see anyone for antenatal care for this pregnancy with THIS BABY? | Yes, No |
|  | Whom did you see? Anyone else? *(select all applicable)* | - Doctor  - Nurse/ midwife  - Auxiliary midwife  - Traditional Birth Attendant  - Community/ village health worker  - Other (specify) |
|  | Where did you receive antenatal care for this pregnancy with THIS BABY? Anywhere else? *(select all applicable)* | - Her home  - Other home  - Government hospital  - Government health centre  - Government health post  - Public sector (other - specify)  - Private hospital/ clinic  - Private (other - specify)  - Other (specify) |
|  | How many weeks or months pregnant were you when you first received antenatal care for this pregnancy with THIS BABY? | Weeks, Months, Don’t know^2^  Numeric integer  (Range: 0-45(weeks), 0-11(months)) |
|  | How many times did you receive antenatal care during this pregnancy with THIS BABY? | Numeric integer  (Range: 0-20) |
| Delivery care | Who assisted with the delivery of THIS BABY? Anyone else? | - Doctor  - Nurse/ midwife  - Auxiliary midwife  - Traditional Birth Attendant  - Relative/ Friend  - Other (specify)  - No-one assisted |
|  | Where did you give birth to THIS BABY? | - Her home  - Other home  - Government hospital  - Government health centre  - Government health post  - Public sector (other - specify)  - Private hospital/ clinic  - Private (other - specify)  - Other (specify) |
|  | How long after THIS BABY was delivered did you stay there?^3^  *If <1 day record in hours*  *If <1 week record in days* | Hours, Days, Weeks, Don’t know  Numeric integer  (Range: 0-24(hours), 0-7(days)) |
|  | Was THIS BABY delivered by caesarean section, that is, did they cut your belly open to take the baby out?^3^ | Yes, No |
|  | When was the decision made to have the caesarean section? Was it before or after your labour pains started?^3^ | Before, After |
| Postnatal care | Did anyone check on your health while you were still in the facility?^3^ | Yes, No |
|  | How long after the delivery did the first check take place?^3^  *If <1 day record in hours*  *If <1 week record in days* | Hours, Days, Weeks, Don’t know  Numeric integer |

^1^ The wording and response options for all questions are the same as in the standard DHS-7 model questionnaire except using ‘THIS BABY’ instead of the name of the child in the case of stillbirths, unless otherwise indicated.

^2^ Standard DHS-7 question gave response in months only.

^3^ Asked only for facility births and due to app programming excluded facility births which took place in locations categorised as ‘other’ – excluding 778 children surviving the neonatal period, 81 neonatal deaths, 57 late gestation stillbirths and 32 early gestation stillbirths born in the 5 years prior to the survey.

## Additional file 2.4 Calculation of survey weights

The analysis was restricted to women interviewed in the FBH+ arm.

***Step 1:*** We calculated the probability of a woman with any pregnancy outcome (livebirth, neonatal death, stillbirth) since 1^st^ January 2012 receiving the pregnancy and birth module. For a woman with a stillbirth or neonatal death since 1^st^ January 2012 the probability of being included was 1, as all women with a neonatal death received these additional questions.

For a woman with a livebirth surviving the neonatal period, the probability of receiving the additional questions varied by HDSS site. The weight was calculated as:

$$\frac{100}{\% of women in FBH+ arm completing pregnancy and birth module}$$

For example, in Bandim, the weight was calculated as 100/28.3.

***Step 2:*** The weight for each individual pregnancy outcome was calculated as the inverse of the probability of the pregnancy outcome being selected for each record. For example, if a woman had two neonatal deaths after 2012, only the more recent neonatal death could be included so the individual sampling weight would be 2/1.

***Step 3:*** The weights calculated under steps 1 and 2 were normalized. First the mean weight of all the selected records was calculated. Then the weights calculated in step 2 were divided by the mean weight to estimate new weights. The mean of new weights is 1.

**References**

1. Alam N, Ali T, Razzaque A, Rahman M, Zahirul Haq M, Saha SK, Ahmed A, Sarder AM, Haider M M, Yunus M *et al*: **Health and Demographic Surveillance System (HDSS) in Matlab, Bangladesh**. *Int J Epidemiol* 2017, **46**(3):809-816.
