# Supplementary material for: Stillbirth maternity care measurement and associated factors in population-based surveys: EN-INDEPTH study
Source: Popul Health Metr. 2021 Feb 8;19(Suppl 1):11. doi: 10.1186/s12963-020-00240-1 (PMC7869205; doi:10.1186/s12963-020-00240-1)

# Additional file 4: Additional results

## Additional file 4.1: Don’t know and missing responses by pregnancy outcomes for selected DHS-7 standard pregnancy and postnatal care questions

|  | Child surviving the neonatal period | | | | Neonatal Deaths | | | | Late gestation stillbirths | | | | Early gestation stillbirths | | | |
| --- | --- | --- | --- | --- | --- | --- | --- | --- | --- | --- | --- | --- | --- | --- | --- | --- |
| QUESTION | Total | Missing | Don't Know N (%) | | Total | Missing | Don't Know N (%) | | Total | Missing | Don't Know N (%) | | Total | Missing | Don't Know N (%) | |
| Did you see anyone for antenatal care for this pregnancy with THIS BABY? | 12620 | 1 | NA |  | 1528 | 1 | NA |  | 1033 | 5 | NA |  | 410 | 2 | NA |  |
| Whom did you see? Anyone else? | 11488 | 0 | NA |  | 1389 | 0 | NA |  | 948 | 0 | NA |  | 305 | 0 | NA |  |
| Where did you receive antenatal care for this pregnancy with THIS BABY? | 11488 | 0 | NA |  | 1389 | 0 | NA |  | 948 | 0 | NA |  | 305 | 0 | NA |  |
| How many weeks or months pregnant were you when you 1st received antenatal care? | 11488 | 0 | 164 | 1.4 | 1389 | 0 | 37 | 2.7 | 948 | 0 | 22 | 2.3 | 305 | 0 | 6 | 2.0 |
| How many times did you receive antenatal care during this pregnancy? | 11488 | 4 | 925 | 8.1 | 1389 | 1 | 117 | 8.4 | 948 | 0 | 88 | 9.3 | 305 | 0 | 22 | 7.2 |
| Who assisted with the delivery of THIS BABY? Anyone else? | 12620 | 2 | NA |  | 1528 | 2 | NA |  | 1033 | 6 | NA |  | 410 | 3 | NA |  |
| Where did you give birth to THIS BABY? | 12620 | 2 | NA |  | 1528 | 1 | NA |  | 1033 | 6 | NA |  | 410 | 3 | NA |  |
| How long after THIS BABY was delivered did you stay there? | 7588 | 0 | 48 | 0.6 | 1000 | 0 | 9 | 0.9 | 706 | 0 | 13 | 1.8 | 232 | 0 | 4 | 1.7 |
| Was THIS BABY delivered by caesarean, that is, did they cut your belly open | 7588 | 0 | NA |  | 1000 | 0 | NA |  | 706 | 0 | NA |  | 232 | 0 | NA |  |
| When was the decision made to have the caesarean section? Was it before or | 1706 | 1 | NA |  | 252 | 0 | NA |  | 163 | 0 | NA |  | 23 | 0 | NA |  |
| Did anyone check on your health while you were still in the facility? | 7588 | 0 | NA |  | 1000 | 0 | NA |  | 706 | 0 | NA |  | 232 | 0 | NA |  |
| How long after delivery did the first check take place?^1^ | 6498 | 0 | 427 | 6.6 | 847 | 0 | 64 | 7.6 | 596 | 0 | 49 | 8.2 | 184 | 0 | 13 | 7.1 |

Green rows= <5% don’t knows; yellow rows= 5-10% don’t knows

^1^  Asked only for facility births and due to app programming excluded facility births which took place in locations categorised as ‘other’ – excluding 778 children surviving the neonatal period, 81 neonatal deaths, 57 late gestation stillbirths and 32 early gestation stillbirths born in the 5 years prior to the survey

## Additional file 4.2: Data errors in timing of postnatal care questions

|  | Children surviving the neonatal period  (n= 6,061) | Neonatal deaths  (n= 775) | Late gestation stillbirths  (n= 545) | Early gestation stillbirths  (n= 170) |
| --- | --- | --- | --- | --- |
| Hours in range | 5,660 | 714 | 491 | 157 |
| Hours out of range | 26 | 1 | 2 | 0 |
| Days in range | 351 | 54 | 47 | 13 |
| Days out of range | 24 | 6 | 5 | 0 |
| Combined response in range | 6,011 | 768 | 538 | 170 |
| Combined response out of range | 50 | 7 | 7 | 0 |
| Combined response out of range (%)* | 0.8 | 0.9 | 1.3 | 0 |

*chi-squared p=0.43

## Additional file 4.3: Comparison of selected maternal care indicators by outcome

|  | Late gestation stillbirths compared to neonatal deaths | Late gestation stillbirths compared to children surviving the neonatal period |
| --- | --- | --- |
|  | p value | p value |
| **Antenatal Care Provider^1^** |  |  |
| Doctor | 0.245 | <0.0001 |
| **Number of ANC visits** |  |  |
| 0 | 0.247 | 0.1583 |
| **Timing of 1st ANC visit^2^** |  |  |
| First Trimester | 0.802 | 0.0135 |
| **Birth attendant^3^** |  |  |
| Skilled provider | 0.085 | <0.0001 |
| **Place of birth** |  |  |
| A home | 0.046 | <0.0001 |
| **Mode of delivery** |  |  |
| Elective caesarean section | 0.188 | 0.0303 |
| Emergency caesarean section | 0.809 | <0.0001 |
| **Length of stay at facility** |  |  |
| 3 or more days | 0.009 | <0.0001 |
| **Postnatal check** |  |  |
| Yes | 0.911 | 0.433 |

^1^ Refers to the highest level provider.

^2^ For categorisation, trimesters were defined as first (<13 weeks, <3 months), second (13 – 27 weeks, 3 – 6 months), third (28 weeks, 7 months onwards).

^3^ In view of potential difficulty of women knowing the skill of her provider, ‘skilled and unskilled’ healthcare provider groups are merged.

## Additional file 4.4: Distribution of age at first antenatal care visit by outcome

|  | Children surviving the neonatal period | Neonatal deaths | Late gestation stillbirths | Early gestation stillbirths |
| --- | --- | --- | --- | --- |
| Total attended ANC | 11488 | 1389 | 948 | 305 |
| Reported age in weeks, n (%) | 425 (3.7) | 38 (2.7) | 32 (3.4) | 6 (2.0) |
| Reported age in months, n (%) | 10,899 (94.9) | 1,314 (94.6) | 894 (94.3) | 293 (96.0) |
| Didn't know age, n (%) | 164 (1.4) | 37 (2.7) | 22 (2.3) | 6 (2.0) |

### **Additional file 4.4A: Distribution of age at first ANC reported in weeks by outcome (n=501)**


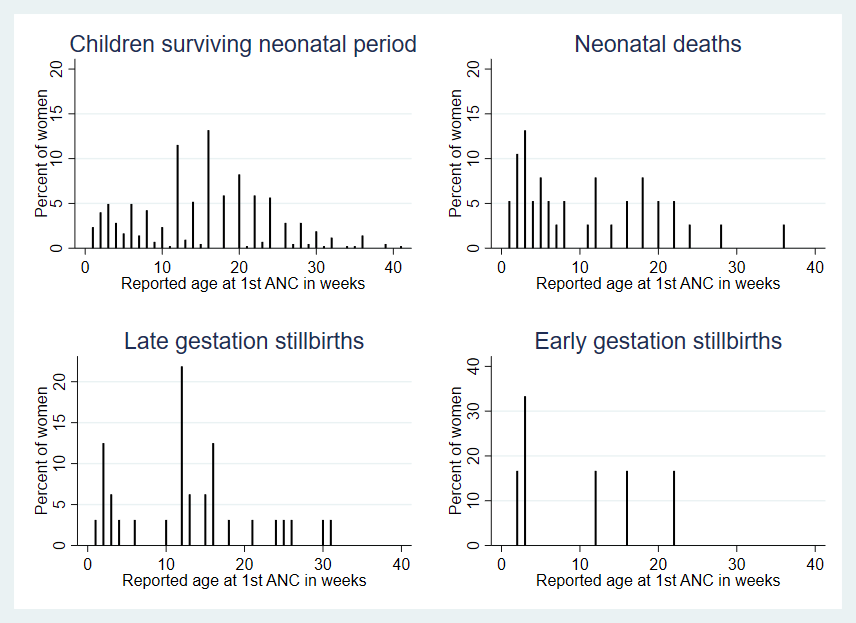


### **Additional file 4.4B: Distribution of age at first ANC reported in months by outcome (n=13,400)**


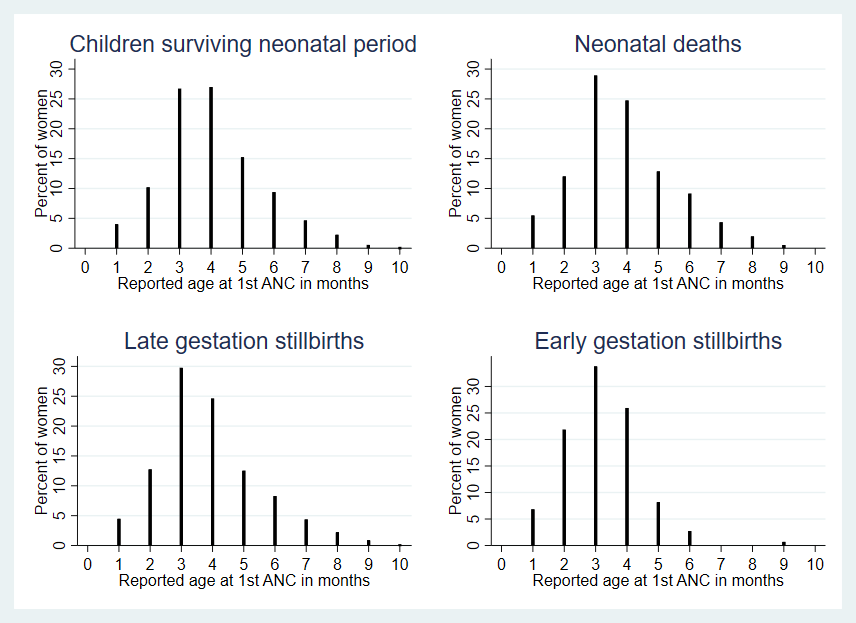


## Additional file 4.5: Distribution of post-delivery length of stay in facility in days


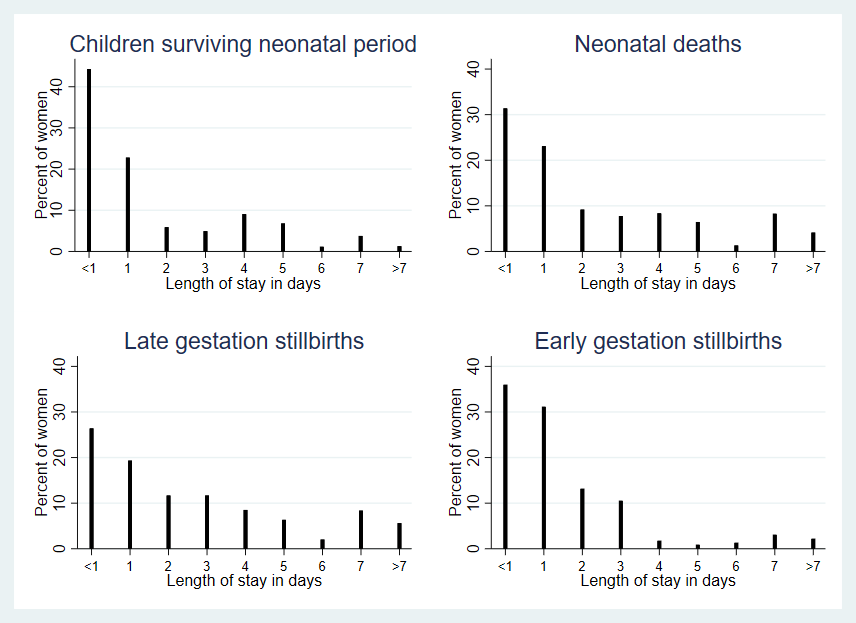


## Additional file 4.6: Distribution of age at first postnatal care visit in hours


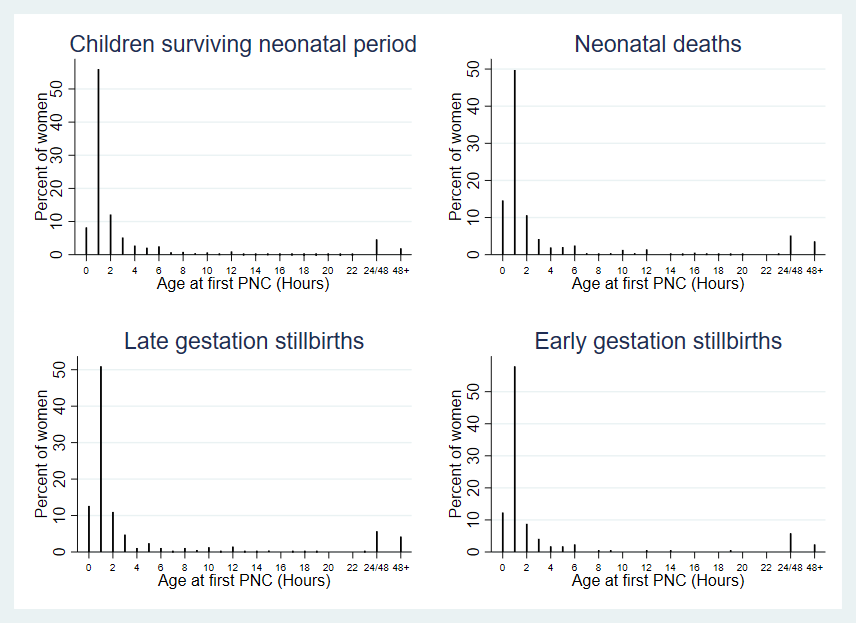

Supplement: Supplementary file 4 — Additional file 4. Additional results. Additional file 4.1: Don’t know and missing responses by pregnancy outcomes for selected DHS-7 standard pregnancy and postnatal care questions. Additional file 4.2: Data errors in timing of postnatal care questions. Additional file 4.3: Comparison of selected maternal care indicators by outcome. Additional file 4.4: Distribution of age at first antenatal care visit by outcome. Additional file 4.4A: Distribution of age at first ANC reported in weeks by outcome (n=501). Additional file 4.4B: Distribution of age at first ANC reported in months by outcome (n=13,400). Additional file 4.5: Distribution of post-delivery length of stay in facility in days. Additional file 4.6: Distribution of age at first postnatal care visit in hours [file 12963_2020_240_MOESM4_ESM.docx]
